# Production and Immunogenicity of FeLV Gag-Based VLPs Exposing a Stabilized FeLV Envelope Glycoprotein

**DOI:** 10.3390/v16060987

**Published:** 2024-06-19

**Authors:** Raquel Ortiz, Ana Barajas, Anna Pons-Grífols, Benjamin Trinité, Ferran Tarrés-Freixas, Carla Rovirosa, Víctor Urrea, Antonio Barreiro, Anna Gonzalez-Tendero, Maria Rovira-Rigau, Maria Cardona, Laura Ferrer, Bonaventura Clotet, Jorge Carrillo, Carmen Aguilar-Gurrieri, Julià Blanco

**Affiliations:** 1 IrsiCaixa, 08916 Badalona, Spain; 2 Doctorate School, Microbiology Department, Universitat Autònoma de Barcelona, 08193 Bellaterra, Spain; 3 Centre for Health and Social Care Research (CESS), Faculty of Medicine, University of Vic-Central University of Catalonia (UVic-UCC), 08500 Vic, Spain; 4 HIPRA, 17170 Amer, Spain; 5 Infectious Diseases Department, Germans Trias I Pujol Hospital, 08916 Badalona, Spain; 6 Germans Trias I Pujol Research Institute (IGTP), 08916 Badalona, Spain; 7 CIBERINFEC, ISCIII, 28029 Madrid, Spain

**Keywords:** Env, vaccine, virus-like particle (VLP), FeLV, SOSIP, veterinary science

## Abstract

The envelope glycoprotein (Env) of retroviruses, such as the Feline leukemia virus (FeLV), is the main target of neutralizing humoral response, and therefore, a promising vaccine candidate, despite its reported poor immunogenicity. The incorporation of mutations that stabilize analogous proteins from other viruses in their prefusion conformation (e.g., HIV Env, SARS-CoV-2 S, or RSV F glycoproteins) has improved their capability to induce neutralizing protective immune responses. Therefore, we have stabilized the FeLV Env protein following a strategy based on the incorporation of a disulfide bond and an Ile/Pro mutation (SOSIP) previously used to generate soluble HIV Env trimers. We have characterized this SOSIP-FeLV Env in its soluble form and as a transmembrane protein present at high density on the surface of FeLV Gag-based VLPs. Furthermore, we have tested its immunogenicity in DNA-immunization assays in C57BL/6 mice. Low anti-FeLV Env responses were detected in SOSIP-FeLV soluble protein-immunized animals; however, unexpectedly no responses were detected in the animals immunized with SOSIP-FeLV Gag-based VLPs. In contrast, high humoral response against FeLV Gag was observed in the animals immunized with control Gag VLPs lacking SOSIP-FeLV Env, while this response was significantly impaired when the VLPs incorporated SOSIP-FeLV Env. Our data suggest that FeLV Env can be stabilized as a soluble protein and can be expressed in high-density VLPs. However, when formulated as a DNA vaccine, SOSIP-FeLV Env remains poorly immunogenic, a limitation that must be overcome to develop an effective FeLV vaccine.

## 1. Introduction

Feline leukemia virus (FeLV) is a simple retrovirus that infects cats along with other felines [1]. While infection is not completely cleared, a small but still significant proportion of infected cats spontaneously control FeLV replication [2]. Although FeLV vaccines are commercially available [3,4,5,6,7], they do not provide a complete protective response and can cause adverse reactions [8]. Therefore, efforts to develop more efficient and safe vaccines continue.

A major challenge in the development of vaccines against retroviruses is the induction of neutralizing antibodies (NAbs). Such antibodies bind to the viral envelope glycoprotein (Env) and prevent viral entry into target cells and the subsequent integration of the provirus into the cellular genome, where it may persist [9]. FeLV Env is a trimeric protein in which each monomer consists of two non-covalently attached subunits; gp70 (SU), involved in receptor binding; and the transmembrane p15E (TM), which plays a major role during the virus/host cell membrane fusion process [10].

Human immunodeficiency virus (HIV) is a complex retrovirus, whose Env is also a trimeric protein complex composed of gp120 (SU) and gp41 (TM) subunits that are held together by weak non-covalent interactions [11,12]. Immunization with unmodified HIV Env proteins has proven to be insufficient to generate protective Nabs; therefore, more sophisticated immunogens have been engineered to present key epitopes targeted by broadly neutralizing antibodies (bNAbs) [13,14]. Since prefusion mature soluble stabilized trimeric HIV Envs were better recognized by bNAbs than by non-NAbs, the initial hypothesis was that this stabilized Env protein might induce neutralizing humoral responses [15,16,17]. Particularly, stable trimeric native-like HIV Env, called SOSIP, is well described [15]. SOSIP-HIV Env trimers contain (i) an engineered disulfide bridge between gp120 and gp41 (mutations A501C and T605C); (ii) a modification of the furin cleavage site by the sequence RRRRRR (R508 to R511) to enhance cleavage; and (iii) a stabilizing I559P point mutation, conferring stability and similar antigenicity as membrane-bound mature viral trimeric Env [15,18,19]. Unfortunately, thus far, the induction of bNAbs after immunization with these variants in combination with adjuvants remains challenging [20,21].

An alternative approach to favor the elicitation of bNAbs is to optimize the antigen delivery [22]. Among different strategies, the multimeric presentation of antigens on the surface of nanoparticles to induce an immune response comparable to natural infections is an attractive option [23,24,25]. Specifically, virus-like particles (VLPs) have been used to express and display HIV Env [26,27]. Our previous work developed a novel approach to optimize antigen delivery by directly fusing an antigen of interest to the N-terminal region of HIV Gag. This strategy allowed the generation of VLPs with a high density of immunogen on its surface with the potential to induce a potent immune response even in the absence of adjuvants and at a low VLP dose [28,29].

By analogy to the HIV Gag-based VLP platform, we have recently generated FeLV Gag-based VLPs as a novel vaccination strategy against this retrovirus [30]. Considering that p15E is one of the targets of neutralizing antibodies [11,31,32,33,34], a fragment of this protein was exposed on the surface of the FeLV Gag-based VLPs. After optimization, the immunogenicity of the selected FeLV Gag-based VLPs candidates was evaluated in two different mouse models, C57BL/6 and BALB/c mice, showing a strong humoral and cellular response against p27 (FeLV Gag capsid), but no response against the p15E antigen [30].

Here, we hypothesized that presenting a stabilized FeLV Env on the surface of VLPs would induce a better neutralizing immune response. By analogy to HIV research on engineered Env, we applied the SOSIP stabilizing approach to FeLV Env, and presented this antigen on the surface of the FeLV Gag-based VLPs. Finally, the immunogenicity of SOSIP-FeLV Env VLPs was evaluated in C57BL/6 mice.

## 2. Materials and Methods

### 2.1. Plasmids 

Fusion proteins were designed by concatenating (from N′ terminus to C′ terminus) a Signal Peptide (SP), the antigen of interest, the FeLV Env membrane-spanning domain (MSD), a GS linker, and the selected FeLV Gag sequence. All the DNA sequences were synthesized at GeneArt (ThermoFisher Scientific, Waltham, MA, USA) and cloned into pcDNA3.4-TOPO vector (ThermoFisher Scientific). They were also subcloned into a pVAX1 vector (ThermoFisher Scientific) using FastDigest KpnI and XhoI restriction enzymes (ThermoFisher Scientific). All the plasmids were transformed into One Shot TOP10 Chemically Competent *E. coli* (Invitrogen) for plasmid DNA amplification. The plasmids were purified in endotoxin-free conditions using the ZymoPure II Plasmid Maxiprep kit (Zymo Research, Irvine, CA, USA) and sterile filtered at 0.22 µm (Millipore, Burlington, MA, USA). The nucleic acid concentration was measured using NanoDrop One/One (ThermoFisher Scientific) and based on the absorbance at 260 nm.

### 2.2. Cell Line, Culture Conditions, and Transfection

The Expi293F cell line (ThermoFisher Scientific) was used for protein and VLP production. The cells were cultured in a Expi293 Expression Medium (Gibco, Waltham, MA, USA) at 37 °C, 8% CO_2_, and under agitation at 125 rpm. All the transfections were performed using an ExpiFectamine transfection kit (Gibco) following the manufacturer’s recommendation. The cells and supernatants were harvested 48 h after transfection. For SOSIP-FeLV soluble protein, the supernatant was harvested 96 h after transfection.

### 2.3. Design, Production, and Purification of SOSIP-FeLV Soluble Protein

The SOSIP-FeLV soluble protein was designed based on a sequence alignment of HIV Env and FeLV-A Env (Uniprot: P04578 and P08359, respectively). Sequence alignment was performed by Pairwise Sequence Alignment using the Needleman/Wunsch algorithm from EMBL-EBI [35]. 

The supernatant of SOSIP-FeLV soluble protein expressed in transiently transfected Expi293F cells was purified by ion metal affinity chromatography (IMAC, Merck, Darmstadt, Germany). The culture supernatant, supplemented with 0.5 M NaCl and 5 mM imidazole, was incubated with Ni SepharoseTM excel beads (Cytiva, Freiburg im Breisgau, Germany) overnight at 4 °C with end-over-end mixing, then transferred to a PolyPrep chromatography column (Bio-Rad, Hercules, CA, USA). Once the Ni Sepharose beads settled by gravity, the column was washed with 1× PBS, 40 mM imidazole, and 0.5 M NaCl. Next, the protein was eluted from the column with 1× PBS and 500 mM imidazole. The eluted sample was concentrated, and buffer exchanged to 1× PBS using a 30 kDa Amicon Ultra-15 Centrifugal Unit (Merck, Darmstadt, Germany). The protein concentration was measured using NanoDrop One/One (ThermoFisher Scientific) based on the absorbance at 280 nm.

### 2.4. Size-Exclusion High-Performance Liquid Chromatography (SE-HPLC)

Size-exclusion high-performance liquid chromatography (SE-HPLC) was performed to assess the molecular weight and oligomerization state of pure SOSIP-FeLV soluble protein. The sample was subjected to SE-HPLC using an Alliance 2695 HPLC System (Waters) and an XBridge BEH 200 Å column (7.8 mm × 300 nm, 3.5 µm) (Waters), flow rate of 0.5 mL/min at room temperature (RT). The standard sample was BEH200 SEC Protein Standard Mix (Waters, Milford, MA, USA). Instrument control, data acquisition, and the compilation of results were performed using the Empower version 2 software (Waters). 

### 2.5. Analysis of VLP and Protein Production

#### 2.5.1. Identification by Western Blot and Coomassie Blue 

The samples (15 µg of total protein) were boiled for 5 min at 95 °C and subjected to electrophoresis in NuPAGE Bis-Tris 4% to 12% acrylamide (ThermoFisher Scientific). The proteins were transferred to a PVDF membrane (Bio-Rad) using the Trans-Blot Turbo Transfer System (Bio-Rad). The membranes were blocked for 1 h at RT with blocking buffer (1× PBS, 0.05% Tween20 (*v*/*v*), and 5% (*w*/*v*) non-fat skim milk powder). The membranes were incubated overnight at 4 °C with the primary antibody anti-FeLV p27 monoclonal antibody [PF12J-10A] (1:2000, Abcam, Cambridge, UK), or anti-gp70 monoclonal antibody [C11D8] (1:2000, ThermoFisher Scientific). After washing with 1× PBS, 0.05% Tween20 (*v*/*v*), and incubation with the secondary antibody, HRP-conjugated AffiniPure Donkey anti-mouse IgG (H+L) (1:10,000, Jackson ImmunoResearch, Baltimore Pike West Grove, PA, USA) was allowed to rest for 1 h. The membranes were developed with SuperSignal West Pico PLUS Chemiluminescent Substrate (ThermoFisher Scientific) according to the manufacturer’s instructions. Chemiluminescence was detected with the ChemiDocTM MP Imaging System (Bio-Rad). For Coomassie staining, the proteins were treated the same as Western blot protocol. Protein gels were incubated in SimpleBlue SafeStain (ThermoFisher Scientific) for 1 h at RT on a rocker. Then, the excess was washed twice with dH_2_O for 1 h and overnight at RT. 

#### 2.5.2. Flow Cytometry Analysis of Recombinant Protein Expression

The transiently transfected Expi293F cells were analyzed by flow cytometry. The VLP-producing cells or soluble protein were stained with the primary antibody, an anti-gp70 monoclonal antibody [C11D8] (1:2000, ThermoFisher Scientific) and AlexaFluor647-labelled goat anti-mouse IgG Fc as a secondary antibody (1:500, Jackson ImmunoResearch), and/or FITC-conjugated anti-FeLV p27 Gag polyclonal antibody (1:100, ThermoFisher Scientific). For cell surface protein expression, the cells were stained as before, then fixed and permeabilized with FIX&PERM (Invitrogen, Carlsbad, CA, USA) and stained with the anti-p27 antibody. For intracellular staining, the cells were fixed and permeabilized before incubation with the antibodies. The cells were acquired using a FACS LSRII Flow Cytometer (BD) with the DIVA Software version 8.0.1.1 (BD, Franklin Lakes, NJ, USA). The flow cytometry results were analyzed using the FlowJo™ v10.6.1 Software (BD). 

#### 2.5.3. Transmission (TEM) and Cryo-Transmission Electron Microscopy (Cryo-EM)

The cells producing VLPs were visualized by TEM. The transiently transfected Expi293F cells were fixed with 0.1 M PBS and 2.5% glutaraldehyde for 2 h at 4 °C, post-fixed with 1% osmium tetroxide with 0.8% potassium ferrocyanide for 2 h, and dehydrated in increasing concentrations of ethanol. Then, the pellets were embedded in epon resin and polymerized at 60 °C for 48 h. Sections of 70 nm in thickness were obtained with a Leica EM UC6 microtome (Leica, Wetzlar, Germany), stained with 2% uranyl acetate and Reynold’s solution (0.2% sodium citrate and 0.2% lead nitrate), and analyzed using a JEM-1400 transmission electron microscope (Jeol Ltd., Tokyo, Japan). All the images were taken at 120 kV. 

VLP extraction and purification were performed following the method described elsewhere [30]. Briefly, VLPs were isolated with lysis buffer (20 mM Phosphate buffer pH 7.4 (Merck), 2 mM EDTA (ThermoFisher Scientific), 2 mM EGTA (Merck), and protease inhibitor (Complete™ ULTRA Tablets EDTA-free, Merck)) containing 0.2% Triton X-100. After detergent removal using Amberlite XAD-4 beads (Merck), the VLP preparations were filtered through a 0.22 μm pore size (Millipore) for sterility and were further purified by ultracentrifugation in a 70% and 30% double sucrose cushion at 40,000× *g* for 2.5 h. Sucrose was removed from the sample by dialysis with Spectra-Por Float-A-Lyzer G2 (Merck) following the manufacturer’s recommendation against 1× PBS. The purified VLP preparations were analyzed by Cryo-EM. The VLPs were deposited on a carbon-coated copper grid and prepared using a Leica EM GP workstation (Leica). The VLPs were observed with a Jeol JEM-2011 (Jeol Ltd.), equipped with a CCD 895 USC4000 camera (Gatan International, Pleasanton, CA, USA). 

### 2.6. Mice Immunization and Immunogenicity Analyses

All the experimental procedures were conducted under the Spanish and European laws and by the guidance of the Institutional Animal Care and Ethics Committee of the Center for Comparative Medicine and Bioimage (CMCiB, Badalona, Spain). They were performed by trained researchers and approved by the regional authorities (Generalitat de Catalunya, Authorization ID: 10583). All the experimental protocols were performed following the principles of the 3Rs, prioritizing the welfare of the animals used in the research. 

DNA immunization was performed in groups of ten six-week-old C57bL/6JOlaHsd mice (n = 50) (Envigo, Indianapolis, IN, USA). Male and female mice were equally represented in each group. Two doses, at weeks 0 and 3, of sterile endotoxin-free DNA were electroporated intramuscularly at the hind leg (20 µg DNA in physiological saline) using a NEPA21 Electroporation System (Nepagene, Chiba, Japan). The electroporation protocol consisted of 8 pulses of 20 ms with a 1 s interval at 60 V. Prior to each immunization, the blood was sampled via facial vein puncture. Serum was recovered from the whole blood after coagulation by centrifugation for 10 min at 4000× *g* and heat-inactivated for 30 min at 56 °C. At week 6, all the animals were euthanized by intracardiac exsanguination, and the blood samples and spleens of each animal were taken for ex vivo immune analysis.

#### 2.6.1. Evaluation of Humoral Response by ELISA 

The levels of antibodies against FeLV p27, p15E, and gp70 in the mouse serum samples were determined by an in-house developed ELISA.

For the anti-FeLV p27 antibody quantification, Nunc MaxiSorp 96-well plates (ThermoFisher Scientific) were coated with 100 ng/well of recombinant FeLV p27 (ProSpec, Rehovot, Israel) and incubated overnight at 4 °C in a wet chamber. Then, the coated plates were blocked with 1× PBS, 1% BSA, and 0.05% Tween20 for 2 h at RT. The mouse anti-p27 monoclonal antibody PF12J-10A (Abcam) was used as standard. The blocked buffer-diluted serum samples (1:100 and 1:1000) were added and incubated overnight at 4 °C in a wet chamber. Total bound IgG was determined with a secondary HRP-conjugated AffiniPure Goat polyclonal anti-mouse IgG (1:10,000) (Jackson ImmunoResearch). The plates were developed with o-Phenylenediamine dihydrochloride (OPD) (Sigma Aldrich, Burlington, MA, USA) and stopped using 2N of H_2_SO_4_ (Sigma Aldrich). The signal was analyzed as the optical density (OD) at 492 nm with noise correction at 620 nm.

For anti-gp70 and anti-p15E antibody quantification, the Nunc MaxiSorp 96-well plates (ThermoFisher Scientific) were coated with 100 ng/well of goat anti-human IgG Fc (Jackson ImmunoResearch) and incubated overnight at 4 °C in a wet chamber. Then, the plates were blocked using 1× PBS, 1% BSA, and 0.05% Tween20 for 2 h at RT. After that, the plates were incubated with SU-huIgG or TM-huIgG Fc-fusion proteins for 2 h at RT. The mouse anti-human IgG1 Fc HP6069 (Merck) was used as standard. The rest of the method followed the same scheme as for anti-FeLV p27 ELISA. TM-huIgG was produced and quantified as described in Ortiz et al. [30]. 

#### 2.6.2. Design and Production of SU-huIgG 

SU-huIgG is a fusion protein designed in-house. The SU fragment (residues 1 to 445 of FeLV Env) was cloned in frame with the Fc portion of human IgG1 (hinge/CH2/CH3) inserted into a pcDNA 3.1 plasmid [36]. It was cloned using the FastDigest KpnI and NheI restriction enzymes (Thermofisher Scientific). The supernatant of the transiently transfected Expi293F cells was used directly for the in-house ELISA. The fusion protein was characterized by Western blot using an HRP-AffiniPure Goat polyclonal anti-human IgG (Jackson ImmunoResearch) and anti-gp70 antibodies and quantified by ELISA as described elsewhere [36]. 

#### 2.6.3. Statistical Analysis

Statistical analyses were performed using Prism 9.0 (GraphPad Software Inc., La Jolla, CA, USA) and R v4.1.1. Comparisons in immunogenicity were tested including undetectable data (under LOD) using the Peto/Peto rank test. For cross-sectional comparisons, multiple comparisons were adjusted using the Benjamini and Hochberg method (FDR). For all the analyses, a *p*-value of less than or equal to 0.05 was considered significant. 

## 3. Results

### 3.1. Generation of a Soluble Stable FeLV Env Trimer

Recently, we have developed a high-density platform of HIV Gag-based VLPs by directly fusing a small antigen of interest to the N-terminal region of HIV Gag through a transmembrane domain and a linker [28,29]. By analogy to HIV Gag-based VLPs, we designed FeLV Gag-based VLPs presenting a fragment of p15E on their surface [30]. Even though these FeLV Gag-based VLP candidates induced a strong cellular and humoral response to FeLV Gag capsid (p27), we failed to generate neutralizing anti-p15E antibodies [30]. Therefore, we decided to expose a more complex Env antigen on the surface of FeLV Gag-based VLPs.

**Figure 1:**
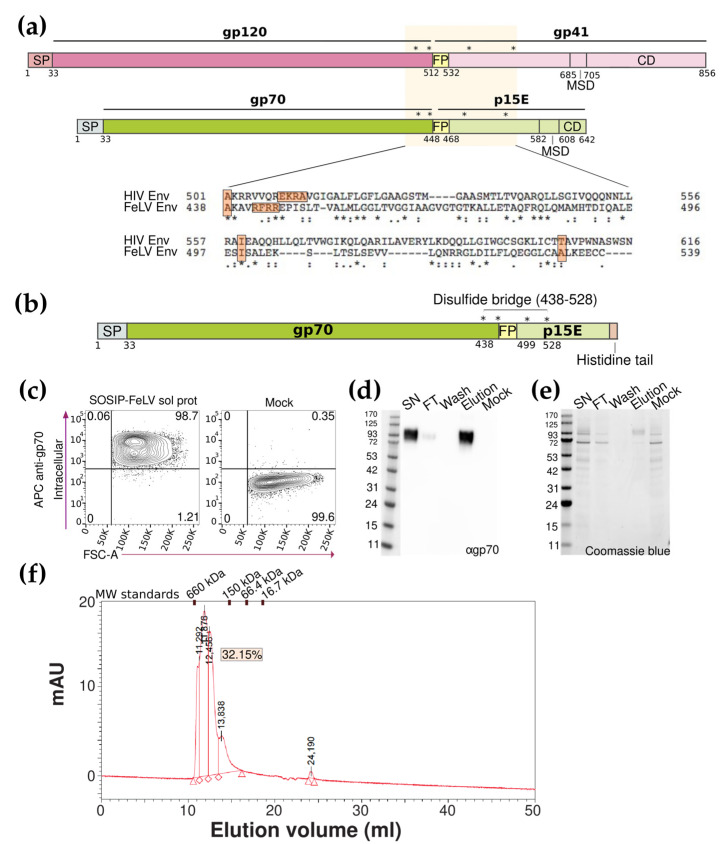
Generation of a soluble stable Env trimer. (**a**) Schematic representation of HIV and FeLV Env glycoproteins, mutations of SOSIP strategy are indicated by asterisks. Alignment between HIV and FeLV Env glycoproteins. (**b**) The schematic representation of the SOSIP FeLV soluble protein. Signal peptide (SP), mutations (indicated by asterisks), disulfide bridge, and histidine tail are denoted. (**c**) Representative flow cytometry panels for the total expression of the SOSIP-FeLV soluble proteins detected with an anti-gp70 antibody. (**d**) The Western blot analysis of the different fractions of the SOSIP-FeLV soluble protein purification developed with an anti-gp70 antibody. (**e**) The Coomassie blue analysis of the different fractions of the SOSIP-FeLV soluble protein purification. (**f**) Chromatogram using the SE-HPLC of the SOSIP-FeLV soluble protein.

Translating the SOSIP approach used for stabilizing HIV Env trimers [15] to FeLV Env, we first aligned the two Env sequences (Figure 1a) and identified in the FeLV Env amino acid sequence the equivalent three-point mutations: A438C, I499P, and A532C; as well as the furin cleavage site (Figure 1a). These mutations were included in a novel SOSIP-FeLV soluble protein design containing the SP of FeLV Env (residues 1 to 33), the full gp70 sequence (residues 34 to 442, with a 6-R string in the furin cleavage site (residues 442 to 445)), a fragment of the extracellular portion of p15E (region 448 to 572), and an 8-histidine tail (Figure 1b). The mutations A438C/A532C and I499P were introduced to stabilize the trimeric FeLV Env protein in its prefusion conformation.

The expression of the novel SOSIP-FeLV soluble protein in the transiently transfected Expi293F cells was analyzed by flow cytometry using an anti-FeLV gp70 antibody, confirming its expression (Figure 1c). The supernatant of the transiently transfected Expi293F cells was recovered 96 h after transfection, and the SOSIP-FeLV soluble protein was purified by nickel affinity chromatography. Different fractions were recovered during the purification process: cell lysate (P), raw culture supernatant (SN), flow-through fraction (FT), wash fraction (wash), and the eluted protein (elution). All the fractions were analyzed by Western blot using an anti-FeLV gp70 antibody, confirming the expression and purification of the protein, which showed an apparent molecular weight of 90 kDa (Figure 1d). The same fractions were analyzed by Coomassie Blue staining showing an enrichment of the SOSIP-FeLV soluble protein in the elution fraction compared to the starting material (SN) (Figure 1e).

As the native FeLV Env protein is a trimer, we assessed the oligomerization state of the purified SOSIP-FeLV soluble protein by size-exclusion chromatography (SEC). The interpolated values of major peaks indicated the presence of multimers in the purified sample. More specifically, trimers, with an apparent molecular weight of 339 kDa, were detected in a proportion of around 32% of the total sample. These data indicate that the SOSIP-FeLV soluble protein is able to oligomerize in trimers, but also in lower and higher order oligomers under our purification conditions (Figure 1f).

### 3.2. Generation of SOSIP-FeLV Gag-Based VLPs 

**Figure 2:**
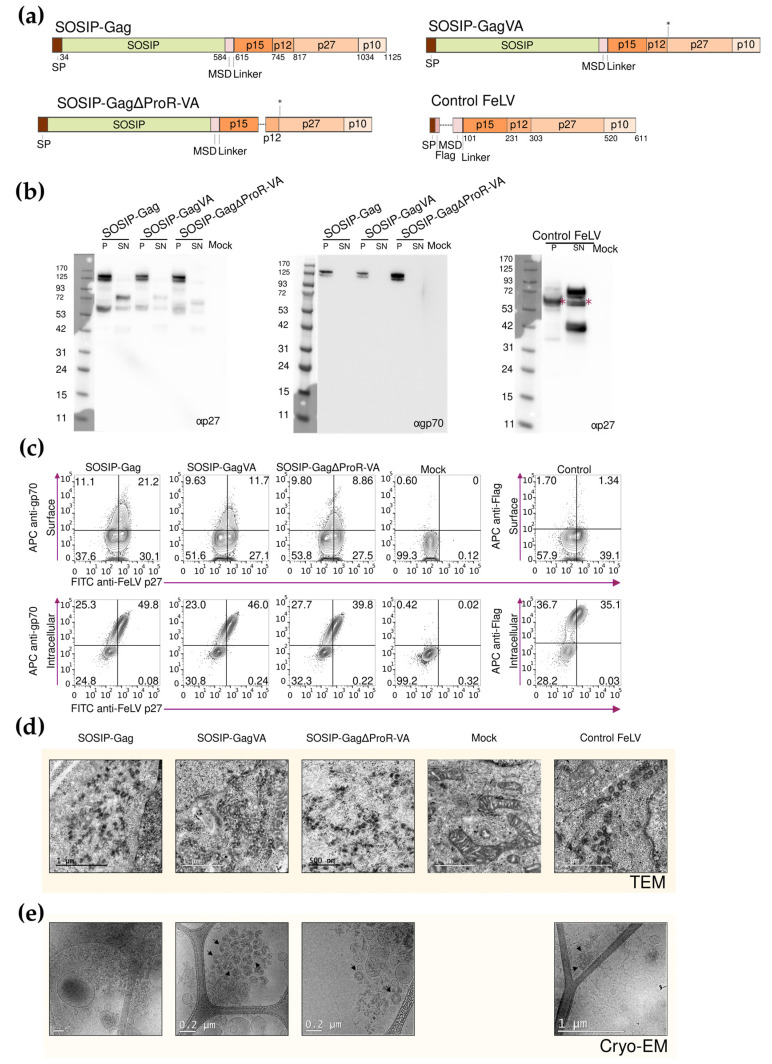
Design and characterization of the SOSIP-FeLV Gag fusion proteins. (**a**) The schematic representation of each fusion protein, asterisks indicate location of the VA mutations in FeLV Gag. (**b**) Western blot was developed with anti-p27 or anti-gp70 antibodies to analyze the expression of the SOSIP-FeLV Gag fusion proteins in the Expi293F cells. Asterisks show estimated molecular weight. (**c**) Representative flow cytometry panels for the extracellular and intracellular detection of the fusion proteins using anti-gp70, anti-Flag, and anti-p27 antibodies. (**d**) TEM images were obtained from the Expi293F cells expressing the indicated FeLV Gag-based VLPs. (**e**) Cryo-EM images were obtained using the purified FeLV Gag-based VLPs.

Having confirmed the expression and oligomerization state of the SOSIP-FeLV protein, we designed, produced, and characterized FeLV Gag-based VLPs exposing the SOSIP-FeLV protein on the surface of the particle. Three different recombinant fusion proteins were designed. All the fusion proteins contained the SP of FeLV Env and the above-described SOSIP-FeLV Env without the Histidine tail, which was replaced by the FeLV Env MSD fused to different forms of FeLV Gag. Based on our previous work to optimize FeLV Gag-based VLP formation [30], the following fusion proteins were tested: (i) SOSIP-Gag, containing wild-type FeLV Gag; (ii) SOSIP-GagVA, containing two mutations at residues L199V and R200A in FeLV Gag; and (iii) SOSIP-GagΔProR-VA, containing a deletion of a late domain-containing region (residues 128 to 153 in FeLV Gag), in addition to the double VA mutation (Figure 2a). Moreover, a control FeLV Gag-based VLP without extracellular FeLV antigens, but with a signal peptide, a Flag-tag, the FeLV Env MSD, and full-length FeLV Gag was designed as a control for immunization assays (control FeLV, Figure 2) [30].

The expression of the recombinant fusion proteins in the transiently transfected Expi293F cells was analyzed by Western blot using anti-FeLV p27 and anti-FeLV gp70 antibodies. A prominent band was observed in cell lysates (P) compared to supernatants (SN, Figure 2b), indicating that the fusion proteins might be retained inside the cells and not secreted to the extracellular media. The expression of the proteins at their expected molecular weight (125 kDa) was only observed in the P lanes (Figure 2b). In addition, the presence of a single band, when the Western blot analysis is performed using an anti-FeLV gp70 antibody, suggests that furin is not acting on the SOSIP-FeLV protein when it is exposed at the surface of the FeLV Gag-based VLPs (Figure 2b).

To explore the possible retention of the fusion proteins inside the cells, we analyzed the transiently transfected Expi293F cells by flow cytometry. The cells were stained at the surface or intracellularly with an anti-FeLV gp70 antibody and intracellularly with an anti-FeLV p27 antibody (Figure 2c). The anti-FeLV gp70 staining was detected mainly in permeabilized cells, confirming that the fusion proteins were retained inside the cells and were not reaching the plasma membrane (Figure 2c), as indicated by the Western blot analysis. Similar results were obtained with control FeLV when staining either the surface or intracellularly with an anti-Flag antibody (Figure 2c).

To confirm the formation of the SOSIP-FeLV Gag-based VLPs, the transiently transfected Expi293F cells were visualized by transmission electron microscopy (TEM). The Expi293F cells transiently transfected with an empty vector were included in the experiment as a control. All three recombinant proteins produced intracellular FeLV Gag-based VLPs, showing a similar morphology and diameter (around 100 nm) to the control FeLV (Figure 2d). The intracellular FeLV Gag-based VLPs were extracted and visualized by cryo-EM. Even though the SOSIP-Gag VLPs were not detected, proper FeLV Gag-based VLPs were detected in the SOSIP-GagVA and SOSIP-Gag∆ProR-VA preparations (Figure 2e). Taken together, these data suggest that SOSIP-GagVA and SOSIP-Gag∆ProR-VA fusion proteins promote the expression of morphologically correct FeLV Gag-based VLPs.

### 3.3. Immunogenicity of FeLV Gag-Based VLPs in C57BL/6 Mice

**Figure 3:**
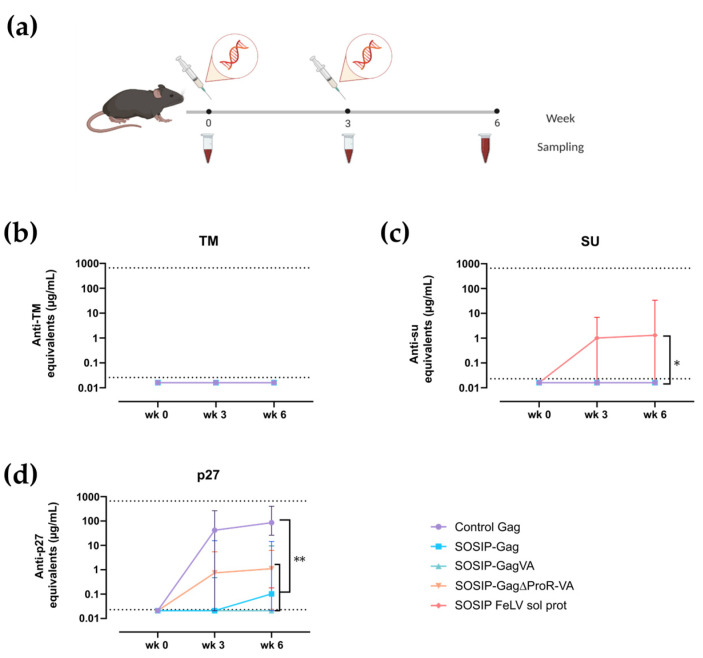
Immunogenicity of the SOSIP-FeLV Gag-based VLPs in C57BL/6. (**a**) The graphical scheme of the experimental procedure. Antibody titers against FeLV TM (**b**), FeLV SU (**c**), and FeLV p27 (**d**) are shown. Data are presented as Median with 95% CI of antibody concentration in mouse sera. Statistical differences were found using the Peto/Peto rank test for week 6 (* *p* < 0.05; ** *p* < 0.01).

The immunogenicity of the SOSIP-FeLV Gag-based VLPs was evaluated in C57BL/6 mice by the intramuscular electroporation of plasmid DNA encoding the fusion proteins SOSIP-Gag, SOSIP-GagVA, SOSIP-Gag∆ProR-VA, and control Gag. Even though the SOSIP-Gag VLPs were not detected by cryo-EM (Figure 2e), the transiently transfected Expi293F cells were able to generate VLPs (Figure 2d). Consequently, this fusion protein was also selected for in vivo DNA immunization. In addition, we included the SOSIP-FeLV soluble protein and the control Gag as control experimental groups. The animals were vaccinated with two doses of plasmid DNA (20 µg/dose) at weeks 0 and 3, and the endpoint of the experiment was determined at week 3 post-second immunization (Figure 3a). Blood samples were taken prior to each immunization and at the endpoint (Figure 3a).

Humoral immune responses were evaluated against FeLV TM, FeLV SU, and FeLV p27 antigens using an in-house ELISA. None of the SOSIP-FeLV Gag-based VLP- or SOSIP-FeLV soluble protein-vaccinated animals generated detectable antibodies against FeLV TM (Figure 3b). In contrast, the analysis of humoral response against FeLV SU showed that only the SOSIP-FeLV soluble protein was able to generate a response in some animals (n = 3/10 positive animals, Figure 3c). Finally, the analysis of humoral responses against FeLV p27 (corresponding to FeLV Gag capsid) showed a readily measurable response in FeLV Gag-based VLP-vaccinated groups (Figure 3d). However, only the control Gag induced a high titer of antibodies against FeLV p27, reaching a plateau after a single dose (Figure 3d). In contrast, the SOSIP-FeLV-expressing VLPs induced a significantly lower response against FeLV p27 (*p*-value = 0.0011, Figure 3d). Taken together, immune responses to FeLV Env are not improved by multivalent presentation, and this protein has a negative impact on anti-Gag responses, suggesting not only a poor immunogenicity but an immunosuppressive effect of FeLV Env and its SOSIP derivative.

## 4. Discussion

A fully protective vaccine against retroviruses, such as HIV or FeLV, is still unavailable. Currently, one of the most promising strategies to improve the immunogenicity of the Env-based subunit vaccines is the modification of the Env sequence to produce stable soluble trimers. SOSIP modifications [22] allow us to produce trimeric HIV Env in a stable prefusion conformation. SOSIP-HIV Env trimers have demonstrated their immunogenic potential in preclinical experiments in non-human primates [37] and have recently reached clinical trials in humans [31]. Therefore, we hypothesized that a SOSIP approach could be also applied to FeLV Env to increase its immunogenicity. Although many efforts to design a FeLV vaccine that induces both humoral and cellular responses have been made [32,33,34,38], currently, three vaccine formulations against FeLV are commercially available: (i) classical inactivated virus vaccines [39,40]; (ii) subunit vaccines based on the FeLV Env subunit gp70 [41]; and (iii) infectious recombinant canarypox virus engineered to express FeLV genes [5,42]. Unfortunately, none of them provide full protection against FeLV infection [43,44,45]. Therefore, new efforts are needed to design more efficient vaccines against this virus. 

Using HIV as a reference, we have designed a SOSIP-FeLV soluble protein. To our knowledge, this study represents the first application of the SOSIP strategy to the FeLV Env. After aligning both Env sequences, we identified the equivalent amino acids and successfully introduced mutations to stabilize the FeLV Env trimer. In vitro analysis confirmed that the SOSIP-FeLV soluble protein is produced with a heterogenous oligomerization profile, including trimers, as well as higher- and lower-order oligomers. Therefore, reducing this heterogeneity may be beneficial for generating a protective and neutralizing response against the viral infection. This can be achieved by making additional stabilizing mutations, modifying the conditions of cell culture and expression, and improving the protocol of purification and the formulation buffer.

Alternatively, to increase the immunogenicity of FeLV Env, it may be beneficial to present the protein at high density in a particulate format, such as on the surface of VLPs. To achieve this, we fused the SOSIP-FeLV Env to FeLV Gag through the FeLV Env MSD. The FeLV Gag was modified with different N-terminal mutations (GagVA and Gag∆ProR-VA) to produce the FeLV Gag-based VLPs more efficiently, as previously described [30]. After ensuring that all the designed fusion proteins properly formed VLPs, we tested the immunogenicity of the soluble SOSIP-FeLV Env trimers in vivo as soluble proteins or as VLPs through DNA immunization. This delivery method is known to induce both humoral and cellular responses [29,46,47], and eliminates the need to produce and purify the immunogens, which can be a challenging task, particularly for VLPs.

Unexpectedly, no response against the SOSIP-FeLV Env subunits, SU or p15E, was observed in any of the VLP-vaccinated groups. Only a minor response against SU was observed for the SOSIP-FeLV soluble protein-vaccinated group. Additionally, the humoral response against the FeLV Gag (p27 subunit) was low or not detected in the SOSIP-FeLV Gag-based VLP-vaccinated groups when compared with the high immunogenicity observed in the control Gag-vaccinated group. Therefore, the humoral responses to FeLV Gag and FeLV Env antigens were lower in the VLP-immunized groups compared to the respective controls (control Gag and SOSIP-FeLV soluble protein). This could be due to various factors. First, DNA vaccination has a Th1 bias, potentially limiting humoral responses [48]. In addition, VLPs can be taken up by endocytosis by surrounding cells, impairing their ability to reach antigen-presenting cells. However, when a similar approach fusing HIV or RSV antigens to retroviral Gag has been tested, excellent immunogenicity results have been obtained [29,47]. One alternative explanation for the low humoral response against the FeLV Gag in the SOSIP-FeLV VLP-vaccinated groups may be the low production of FeLV Gag-based VLPs in vivo. It is worth noting that the Env protein of retroviruses is a large and glycosylated protein, with a mass of approximately 90 kDa for FeLV Env monomer. This is in contrast with other simpler immunogens, such as the Flag Tag used for the control Gag group or p15E FeLV Gag-based VLPs [30]. Therefore, the formation of Env FeLV Gag-based VLPs is a complex process that may result in low VLP production efficacy in vivo and therefore a low humoral response after two vaccine doses. Finally, a potential reason for the low humoral response to FeLV Env could be the immunosuppressive activity of this glycoprotein. Although the primary immunosuppressive domain of p15E [49] was mutated in our FeLV Env design, other specific sequences, or a glycosylation pattern on the FeLV Env could limit its immunogenicity. Similarly, an earlier study, which integrated SU expression within a recombinant vaccinia virus vector, also failed to produce neutralizing antibodies against FeLV Env [38]. Taken together, these results suggest a poor immunogenicity or an immunosuppressive function of FeLV Env. Our data showing that SOSIP-FeLV Env presented on the surface of FeLV Gag-based VLPs was unexpectedly less immunogenic than the soluble form, point to the immunosuppressive activity as a plausible hypothesis. However, this observation could be related to the fact that SOSIP-FeLV Env is not properly cleaved by furin when expressed on the surface of FeLV Gag-based VLPs. As a result, the SOSIP-FeLV Env protein would retain its immature conformation, which may render it poorly immunogenic, as is the case with HIV [15]. Additionally, the high density of Env on the VLP surface might obstruct access to the membrane-fusion subunit, p15E, even though it contains conserved epitopes.

In summary, we have described a new potential FeLV Env immunogen applying the SOSIP strategy described for HIV. The preliminary characterization of its expression and immunogenicity using a DNA-based approach suggests that further optimization in both aspects is required to evaluate its potential. Particularly, protein-based approaches after defining an upstream and downstream production process and an optimal immunogenic delivery (including adjuvants) need to be tested. In addition, the definition of immunosuppressive activity of FeLV Env deserves further investigation.

## Data Availability

The original contributions presented in the study are included in the article, further inquiries can be directed to the corresponding authors.
